# Global disease burden and trends of leukemia attributable to occupational risk from 1990 to 2019: An observational trend study

**DOI:** 10.3389/fpubh.2022.1015861

**Published:** 2022-11-14

**Authors:** Yuanfei Shi, Can Chen, Yamei Huang, Yi Xu, Dandan Xu, Huafei Shen, Xiujin Ye, Jie Jin, Hongyan Tong, Yue Yu, Xinyi Tang, Azhong Li, Dawei Cui, Wanzhuo Xie

**Affiliations:** ^1^Department of Hematology, The First Affiliated Hospital, Zhejiang University School of Medicine, Hangzhou, China; ^2^State Key Laboratory for Diagnosis and Treatment of Infectious Diseases, National Clinical Research Center for Infectious Diseases, Collaborative Innovation Center for Diagnosis and Treatment of Infectious Diseases, The First Affiliated Hospital, Zhejiang University School of Medicine, Hangzhou, China; ^3^Department of Pathology and Pathophysiology, Medical School of Southeast University, Nanjing, China; ^4^Department of Blood Transfusion, The First Affiliated Hospital, Zhejiang University School of Medicine, Hangzhou, China; ^5^International Health Care Center, The First Affiliated Hospital, Zhejiang University School of Medicine, Hangzhou, China; ^6^Department of Quantitative Health Science, Mayo Clinic, Rochester, MN, United States; ^7^Mayo Clinic, Rochester, MN, United States; ^8^Zhejiang Blood Center, Hangzhou, China

**Keywords:** leukemia, AML, ALL, global burden disease, death rate

## Abstract

**Background:**

Leukemia caused by occupational risk is a problem that needs more attention and remains to be solved urgently, especially for acute lymphoid leukemia (ALL), acute myeloid leukemia (AML), and chronic lymphoid leukemia (CLL). However, there is a paucity of literature on this issue. We aimed to assess the global burden and trends of leukemia attributable to occupational risk from 1990 to 2019.

**Methods:**

This observational trend study was based on the Global Burden of Disease (GBD) 2019 database, the global deaths, and disability-adjusted life years (DALYs), which were calculated to quantify the changing trend of leukemia attributable to occupational risk, were analyzed by age, year, geographical location, and socio-demographic index (SDI), and the corresponding estimated annual percentage change (EAPC) values were calculated.

**Results:**

Global age-standardized DALYs and death rates of leukemia attributable to occupational risk presented significantly decline trends with EAPC [−0.38% (95% CI: −0.58 to −0.18%) for DALYs and −0.30% (95% CI: −0.45 to −0.146%) for death]. However, it was significantly increased in people aged 65–69 years [0.42% (95% CI: 0.30–0.55%) for DALYs and 0.38% (95% CI: 0.26–0.51%) for death]. At the same time, the age-standardized DALYs and death rates of ALL, AML, and CLL were presented a significantly increased trend with EAPCs [0.78% (95% CI: 0.65–0.91%), 0.87% (95% CI: 0.81–0.93%), and 0.66% (95% CI: 0.51–0.81%) for DALYs, respectively, and 0.75% (95% CI: 0.68–0.82%), 0.96% (95% CI: 0.91–1.01%), and 0.55% (95% CI: 0.43–0.68%) for death], respectively. The ALL, AML, and CLL were shown an upward trend in almost all age groups.

**Conclusion:**

We observed a substantial reduction in leukemia due to occupational risks between 1990 and 2019. However, the people aged 65–69 years and burdens of ALL, AML, and CLL had a significantly increased trend in almost all age groups. Thus, there remains an urgent need to accelerate efforts to reduce leukemia attributable to occupational risk-related death burden in this population and specific causes.

## Introduction

Cancer has the highest mortality rate among all human diseases (1). The World Health Organization (WHO) classifies tumors based on evidence that tumors occur in various organ systems. It is the global standard for diagnosis, research, cancer registration, and public health monitoring (2). Some kinds of cancer grow rapidly, while others grow slowly. Most kinds of leukemia progress quickly. Hematological tumors can be divided into three different categories, namely, leukemia, lymphoma, and myeloma (3). Among them, leukemia can be further divided into acute myeloid leukemia (AML), acute lymphoid leukemia (ALL), chronic lymphoid leukemia (CLL), chronic myeloid leukemia (CML), and other leukemia (4). In this study, we found that AML and ALL had higher DALY rates and death rates compared with other kinds.

Acute myeloid leukemia is the most common in elderly patients, but the incidence rate in young people is also increasing every year (5), with an incidence of over 20,000 cases per year in the United States alone (6). It has been estimated that ~21,450 adults (11,650 men and 9,800 women) will be diagnosed with AML in 2019 (7). Of all subtypes of leukemia, AML has the highest mortality rate (62%) (7). The excessive accumulation of immature hematopoietic cells in blood and bone marrow, gene mutations, genetics, and other factors will lead to the occurrence of tumors (8). ALL is the most common subtype of childhood leukemia. ALL have a high mortality rate due to excessive accumulation of immature lymphocytes in the peripheral blood and bone marrow (9, 10).

Occupational exposure has been related to higher risks of several kinds of cancer (11, 12). It has brought tremendous health and economic burden for people all over the world (13). However, insufficient attention has been paid to the detection, diagnosis, and monitoring of occupational exposure and its associated diseases, especially leukemia. It is well-accepted that occupational exposure to formaldehyde and benzene causes leukemia (14, 15). The researchers compared occupational exposure to formaldehyde with the risk of leukemia in community-based case-control studies. The proportion of leukemia in occupationally exposed cohorts will increase significantly (16). In 2009, the International Agency for Research on Cancer (IARC) regarded formaldehyde as a risk factor for leukemia (17, 18). Researchers often focus on the relationship between formaldehyde and general lymphohematopoietic cancer or leukemia, but the issue of occupational exposure has not attracted their attention (16, 19, 20).

The Global Burden of Disease (GBD) study was originally authorized by the World Bank and added to the landmark World Development Report 1993 (21). Since 1990, GBD has made the most comprehensive efforts to systematically monitor and master the world's health problems (22). We used the GBD database to analyze leukemia attributable to occupational risk incidences and deaths in the general population by sex, social development index (SDI), and reason for the 1990–2019 period at regional and global levels. We aimed to provide valuable insights into data-based healthcare regimens and provide a better understanding of the global burden of leukemia attributable to occupational risk as an important complement to previous GBD studies.

## Methods

### Data sources

Data on the burden of leukemia attributable to occupational risk were downloaded from the Global Health Data Exchange GBD Results Tool (http://ghdx.healthdata.org/gbd-results-tool), including death rates and disability-adjusted life years (DALYs). GBD values were reported as estimated values with 95% uncertainty intervals (UIs), and a posterior distribution was used to calculate the 25th and 975th ranked estimates from random 1,000 draws (23). Information such as the socio-demographic index (SDI) and corresponding age-standardized rates was also downloaded from this website for the following correlation analysis. Based on the SDI, 204 countries and territories were divided into five super regions, namely, low, low-middle, middle, middle, and high SDI (24, 25). According to GBD 2019, SDI is an indicator of a country's level of health development, based on fertility rates for women under 25 and total fertility rates for men, education attainment among those 15 years of age and older, and 10-year lag-distributed average individual incomes (26).

The SDI values range between 0 and 1, which reflect the degree of social development. Our research is compliant with the Guidelines for Accurate and Transparent Health Estimates Reporting.

### Definitions

The occupational risk was defined as patients' long-term exposure to carcinogenic factors in the working environment. These kinds of occupational carcinogenic factors include chemical, physical, and biological (27). The DALYs by age, sex, year, and region were collected from GBD 2019. The DALYs is a summary measure that quantifies the overall burden of disease (28, 29).

### Statistical analysis

We estimated the number of deaths or DALYs, age-standardized rate DALYs, and deaths to quantify leukemia attributable to occupational risk by age, year, and region.

The estimated annual percentage change (EAPC) was calculated to quantify the trends of burdens of leukemia attributable to occupational risk from 1990 to 2019. The regression model was used to fit the age-standardized rate (ASR), that is, ln (ASR) = α + βX + ε, where y stands for the burden rate and x for the calendar year. EAPC was calculated by 100 × [exp (β)−1], and its 95% confidence interval (CI) could also be calculated from the model (24, 25, 30). With the EAPC value and its 95% CI above zero, the corresponding age-standardized rate (ASR) was in an upward trend and *vice versa* (31). Moreover, to gain a better understanding of the relationship between the EAPC of ASR and possible facts, a local weighted scatter plot smoothing regression was used to display more detailed information (24, 25). All statistical analyses were done using R (version 3.6.0).

## Results

### The distribution and its change trend of leukemia attributable to occupational risk

**Figure 1:**
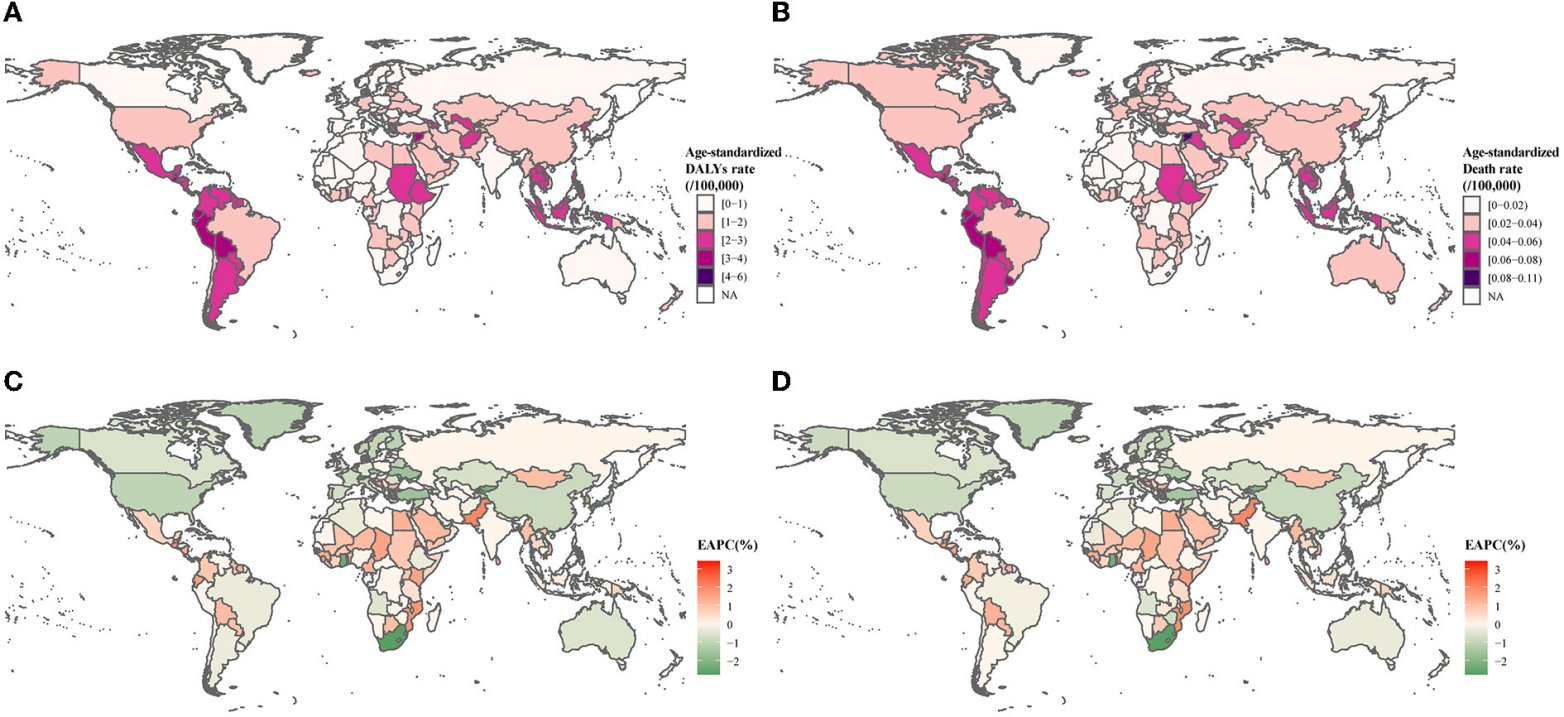
Age-standardized DALY and death rates in 2019 for leukemia attributable to occupational risk. **(A)** Age-standardized disability-adjusted life years rate. **(B)** Age-standardized death rates. **(C)** Estimated annual percent change of disability-adjusted life years rate. **(D)** Estimated annual percent change of death rates.

The global age-standardized DALYs and death rates of leukemia attributable to occupational risk were 1.5609, 95% UI: 0.7676–2.2942 for DALYs and 0.0326, 95% UI: 0.0161–0.0481 for deaths in 1990 and 1.3986, 95% UI: 0.669–2.0648 for DALYs and 0.0299, 95% UI: 0.0144–0.0445 for deaths in 2019. Male individuals had higher age-standardized rates than female individuals in leukemia attributable to occupational risk. Global age-standardized DALYs and death rates presented significantly decline trends with EAPCs [−0.38% (95% CI: −0.58 to −0.18%) for DALYs and −0.30% (95% CI: −0.45 to −0.146%) for death] (Table 1). The highest age-standardized DALYs and death rates were observed in the regions of Andean Latin America, Central Latin America, and the Caribbean, whereas the lowest age-standardized incidence rates were seen in Southern sub-Saharan Africa (Figures 1A,B). The most pronounced increase in age-standardized DALYs and death rates was detected in the regions of Latin America and the Caribbean (Andean Latin America, Central Latin America, and the Caribbean), sub-Saharan Africa (Eastern sub-Saharan Africa and Western sub-Saharan Africa), and Southeast Asia and Oceania (Figures 1C,D, Table 1).

**Table 1:** The number and age-standardized rate of DALYs and death of leukemia attributable to occupational risk in 1990 and 2019.

	**DALYS (95% UI)**					**Deaths (95% UI)**				
	**1990**		**2019**		**EAPC (95% CI)**	**1990**		**2019**		**EAPC (95% CI)**
	**Number**	**Age-Standardized rate**	**Number**	**Age-Standardized rate**		**Number**	**Age-Standardized rate**	**Number**	**Age-Standardized rate**	
Global	80,359 (39,266–118,130)	1.5609 (0.7676–2.2942)	113,715 (54,505–167,831)	1.3986 (0.669–2.0648)	−0.38% (95% CI: −0.58 to −0.18%)*	1,612 (792–2,369)	0.0326 (0.0161–0.0481)	2,455 (1,181–3,645)	0.0299 (0.0144–0.0445)	−0.3% (95% CI: −0.45 to −0.14%)*
**Gender**										
Male	46,410 (22,691–68,190)	1.7925 (0.8719–2.6295)	66,189 (32,299–98550)	1.6267 (0.7944–2.4214)	−0.33% (−0.41 to −0.24%)*	932 (460–1,370)	0.0378 (0.0186–0.0557)	1,425 (699–2,131)	0.035 (0.0172–0.0524)	−0.33% (−0.53 to −0.14%)*
Female	33,949 (17,064–51,519)	1.3279 (0.6663–2.011)	47,526 (21,915–71,940)	1.1703 (0.5417–1.7683)	−0.44% (−0.69 to −3.33%)*	680 (340–1,032)	0.0275 (0.0137–0.0416)	1,030 (478–1,563)	0.025 (0.0116–0.0379)	−0.27% (−0.30 to −0.23%)*
**Age group**										
15–49 year	69,226 (34,198–101,973)	2.5524 (1.2609–3.7598)	90,522 (42,924–133,843)	2.3,004 (1.0908–3.4013)	−0.36% (−0.58 to −0.13%)*	1,229 (607–1,808)	0.0453 (0.0224–0.0667)	1,635 (783–2,420)	0.0416 (0.0199–0.0615)	−0.3% (−0.51 to −0.09%)*
50–69 year	9,616 (4,607–14,350)	1.4097 (0.6754–2.1037)	18,828 (8,926–28,358)	1.3654 (0.6473–2.0565)	−0.11% (−0.16 to −0.06%)*	302 (144–451)	0.0442 (0.0211–0.0661)	589 (280–887)	0.0427 (0.0203–0.0643)	−0.11% (−0.19 to −0.04%)*
70+ year	1,517 (608–2,360)	0.7528 (0.3015–1.1712)	4,365 (1,892–6,819)	0.9413 (0.408–1.4706)	0.79% (0.69–0.88%)*	81 (33–126)	0.0402 (0.0162–0.0624)	230 (101–358)	0.0497 (0.0218–0.0773)	0.74% (0.64–0.84%)*
**SDI region**										
High SDI	12,245 (3,626–20,127)	1.3381 (0.3967–2.1986)	13,025 (4,307–21,114)	1.0821 (0.3572–1.7444)	−0.71% (−0.82 to −0.61%)*	282 (83–463)	0.03 (0.0088–0.0493)	343 (108–558)	0.0251 (0.0082–0.0408)	−0.6% (−0.64 to −0.55%)*
High-Middle SDI	21,953 (11,034–32,036)	1.8066 (0.9141–2.6395)	25,292 (12,559–37,288)	1.5348 (0.7621–2.2598)	−0.55% (−0.81 to −0.28%)*	442 (223–646)	0.037 (0.0187–0.0541)	552 (272–817)	0.0318 (0.0158–0.0473)	−0.51% (−0.74 to −0.28%)*
Middle SDI	30,894 (14,963–45,905)	1.8519 (0.8966–2.7548)	44,229 (20,790–65,558)	1.6778 (0.7894–2.4881)	−0.34% (−0.59 to −0.09%)*	590 (286–876)	0.0375 (0.0182–0.0557)	925 (435–1383)	0.0348 (0.0164–0.052)	−0.22% (−0.4 to −0.03%)*
Low-Middle SDI	11,144 (5,399–16,808)	1.1395 (0.5449–1.7147)	20,970 (9,883–31,866)	1.1817 (0.5556–1.7971)	0.13% (−0.05–0.31%)	216 (103–325)	0.0235 (0.0113–0.0353)	432 (202–659)	0.0252 (0.0119–0.0385)	0.24% (0.09–0.38%)*
Low SDI	4,081 (1,876–6,554)	1.019 (0.4688–1.6285)	10,119 (4,754–15,494)	1.1048 (0.5093–1.6935)	0.28% (0.2–0.37%)*	81 (37–130)	0.0219 (0.0102–0.0351)	201 (93–309)	0.0241 (0.011–0.0372)	0.33% (0.24–0.42%)*
**Type of cause**										
Acute lymphoid leukemia	12,017 (5,800–18,336)	0.223 (0.1069–0.34)	22,397 (10,592–33,563)	0.2788 (0.1318–0.418)	0.78% (0.65–0.91%)*	218 (105–334)	0.0042 (0.002–0.0064)	419 (198–633)	0.0052 (0.0025–0.0078)	0.75% (0.68–0.82%)*
Acute myeloid leukemia	15,418 (7,407–23,786)	0.3035 (0.1446–0.468)	31,670 (15,108–47,562)	0.3891 (0.185–0.5846)	0.87% (0.81–0.93%)*	321 (152–498)	0.0066 (0.0031–0.0103)	712 (346–1080)	0.0087 (0.0042–0.0131)	0.96% (0.91–1.01%)*
Chronic lymphoid leukemia	2,637 (1,247–4,046)	0.0572 (0.0269–0.0877)	5,710 (2,703–8,702)	0.0687 (0.0324–0.1047)	0.66% (0.51–0.81%)*	70 (32–108)	0.0016 (0.0007–0.0025)	155 (74–237)	0.0019 (0.0009–0.0028)	0.55% (0.43–0.68%)*
Chronic myeloid leukemia	10,354 (4,989–15,725)	0.2065 (0.0996–0.3129)	13,455 (6,353–20,693)	0.1652 (0.078–0.2542)	−0.74% (−0.89 to −0.6%)*	216 (105–327)	0.0045 (0.0022–0.0068)	285 (132–438)	0.0035 (0.0016–0.0053)	−0.86% (−1.03 to −0.69%)*
Other leukemia	39,933 (19,388–60,206)	0.7709 (0.3764–1.1628)	40,483 (19,388–59,947)	0.4969 (0.2374–0.736)	−1.51% (−1.7 to −1.32%)*	787 (380–1182)	0.0158 (0.0076–0.0236)	884 (425–1316)	0.0108 (0.0052–0.016)	−1.33% (−1.48 to −1.19%)*
**Southeast Asia, east Asia, and Oceania**										
Southeast Asia	8,013 (3,908–12,237)	1.8404 (0.8829–2.8079)	14,872 (6,972–22,458)	2.0371 (0.9542–3.0779)	0.36% (0.14–0.58%)*	153 (74–233)	0.0377 (0.0183–0.0573)	310 (146–470)	0.043 (0.0202–0.0652)	0.47% (0.31–0.63%)*
East Asia	28,451 (14,364–42,465)	2.1654 (1.0907–3.2396)	29,187 (14,636–44,374)	1.7044 (0.8521–2.5867)	−0.83% (−1.33 to −0.34%)*	543 (271–809)	0.0432 (0.0218–0.0644)	616 (312–934)	0.034 (0.017–0.0514)	−0.84% (−1.23 to −0.44%)*
Oceania	84 (38–136)	1.4493 (0.6591–2.3427)	206 (86–365)	1.6147 (0.672–2.8499)	0.39% (0.31–0.47%)*	2 (1–3)	0.0292 (0.0131–0.0473)	4 (2–7)	0.033 (0.0137–0.0575)	0.43% (0.36–0.51%)*
**Sub-Saharan Africa**										
Western Sub-Saharan Africa	1,301 (630–1,983)	0.8805 (0.4208–1.3465)	3,323 (1,452–5,392)	0.9098 (0.3995–1.4641)	0.11% (−0.01–0.24%)	26 (12–39)	0.0187 (0.0089–0.0287)	65 (28–104)	0.0195 (0.0085–0.031)	0.14% (0–0.27%)*
Central Sub-Saharan Africa	456 (213–725)	1.0641 (0.5–1.7038)	1,122 (520–1,850)	1.0326 (0.4854–1.7219)	−0.09% (−0.31–0.13%)	9 (4–14)	0.0223 (0.0104–0.0353)	21 (10–36)	0.0216 (0.01–0.0358)	−0.1% (−0.31–0.11%)
Southern Sub-Saharan Africa	693 (316–1,063)	1.5077 (0.6848–2.3022)	698 (304–1,109)	0.8289 (0.3623–1.2965)	−2.14% (−2.61 to −1.67%)*	13 (6–20)	0.0309 (0.0143–0.047)	13 (6–21)	0.0165 (0.0072–0.0256)	−2.11% (−2.6 to −1.63%)*
Eastern Sub-Saharan Africa	1,892 (836–3,251)	1.3829 (0.6181–2.3596)	4,615 (2,009–7,528)	1.4389 (0.621–2.3458)	0.14% (0–0.27%)*	37 (17–64)	0.03 (0.0137–0.0508)	92 (40–150)	0.0323 (0.0139–0.0531)	0.26% (0.11–0.41%)*
South Asia	7,959 (3,829–11,931)	0.8339 (0.4039–1.2503)	16,167 (7,637–25,073)	0.8775 (0.415–1.3532)	0.17% (−0.11–0.45%)	158 (76–237)	0.0176 (0.0085–0.0266)	337 (157–518)	0.019 (0.0089–0.0292)	0.25% (−0.01–0.51%)
**Latin America and Caribbean**										
Tropical Latin America	2,759 (1,283–4,032)	1.9048 (0.8934–2.7924)	4,344 (1,981–6544)	1.76 (0.8031–2.6522)	−0.26% (−0.39 to −0.13%)*	54 (25–79)	0.0397 (0.0187–0.0583)	94 (42–142)	0.0378 (0.0171–0.0572)	−0.15% (−0.27 to −0.03%)*
Caribbean	548 (262–816)	1.616 (0.767–2.4065)	990 (449–1,511)	2.0017 (0.907–3.0462)	0.76% (0.61–0.91%)*	11 (5–16)	0.0342 (0.0164–0.051)	22 (10–33)	0.0431 (0.0196–0.0657)	0.82% (0.68–0.96%)*
Andean Latin America	863 (405–1,355)	2.5525 (1.1909–3.9913)	2,123 (939–3,345)	3.2553 (1.4393–5.1235)	0.81% (0.11–1.51%)*	17 (8–26)	0.0528 (0.0248–0.0825)	43 (19–69)	0.0683 (0.0307–0.1079)	0.86% (0.19–1.54%)*
Central Latin America	3,386 (1,654–4,978)	2.2682 (1.1108–3.3323)	7,057 (3,264–10,531)	2.6878 (1.2418–4.0114)	0.61% (0.33–0.89%)*	63 (31–93)	0.0457 (0.0225–0.0672)	142 (66–214)	0.0548 (0.0255–0.0825)	0.64% (0.38–0.89%)*
North Africa and Middle East	4,688 (2,220–7,329)	1.6619 (0.7832–2.5851)	10,081 (4,560–15,281)	1.5789 (0.71–2.3953)	−0.17% (−0.27 to −0.07%)*	94 (44–145)	0.0359 (0.0169–0.0555)	210 (94–318)	0.0346 (0.0154–0.0525)	−0.13% (−0.21 to −0.04%)*
**Central Europe, eastern Europe, and central Asia**										
Central Europe	1,275 (374–2,116)	0.9737 (0.2878–1.6158)	1,252 (332–2,154)	0.9031 (0.2423–1.5514)	−0.27% (−0.44 to −0.11%)*	29 (8–48)	0.0213 (0.0062–0.0353)	33 (9–58)	0.0211 (0.0056–0.0365)	−0.04% (−0.19–0.12%)
Central Asia	1,443 (653–2,159)	2.1779 (0.9832–3.2557)	1,936 (834–2,968)	1.9269 (0.8316–2.958)	−0.41% (−0.65 to −0.17%)*	27 (12–41)	0.0432 (0.0195–0.0646)	38 (16–59)	0.0388 (0.0168–0.0597)	−0.35% (−0.61 to −0.09%)*
Eastern Europe	3,077 (830–5,227)	1.257 (0.3367–2.1329)	2,297 (593–3,982)	0.965 (0.2501–1.6661)	−0.83% (−1.54 to −0.12%)*	66 (18–113)	0.0263 (0.007–0.0447)	53 (13–93)	0.0205 (0.0053–0.0359)	−0.77% (−1.24 to −0.29%)*
**High-income regions**										
High-Income North America	4,526 (1,240–7,650)	1.4366 (0.3921–2.4279)	4,584 (1,182–7,868)	1.0589 (0.2726–1.8226)	−1.03% (−1.29 to −0.76%)*	108 (29–182)	0.0336 (0.0091–0.0566)	127 (33–219)	0.0261 (0.0067–0.0448)	−0.83% (−0.95 to −0.7%)*
High-Income Asia Pacific	2,576 (742–4,313)	1.3413 (0.3881–2.2398)	1,868 (509–3,158)	0.8374 (0.2295–1.4079)	−1.65% (−1.88 to −1.42%)*	56 (16–94)	0.0284 (0.0081–0.0476)	50 (13–85)	0.0186 (0.005–0.0315)	−1.45% (−1.63 to −1.28%)*
Australasia	220 (63–370)	0.9914 (0.2819–1.6638)	301 (78–513)	0.8674 (0.2243–1.4794)	−0.43% (−0.6 to −0.26%)*	5 (1–9)	0.0232 (0.0066–0.0392)	8 (2–14)	0.0215 (0.0056–0.0369)	−0.24% (−0.4 to −0.07%)*
Western Europe	4,925 (1,365–8,254)	1.1406 (0.3169–1.9094)	4,950 (1,282–8,350)	0.9437 (0.2439–1.5911)	−0.68% (−0.85 to −0.51%)*	116 (32–196)	0.0254 (0.007–0.0427)	136 (35–232)	0.022 (0.0056–0.0375)	−0.52% (−0.64 to −0.4%)*
Southern Latin America	1,225 (587–1,797)	2.5276 (1.2148–3.7119)	1,743 (787–2,590)	2.4186 (1.0947–3.5928)	−0.17% (−0.29 to −0.06%)*	26 (13–38)	0.0541 (0.0261–0.0795)	39 (18–58)	0.0526 (0.0236–0.0783)	−0.12% (−0.22 to −0.01%)*

The asterisks represent statistically significant.

### Impact of occupational risk on each leukemia

**Figure 2:**
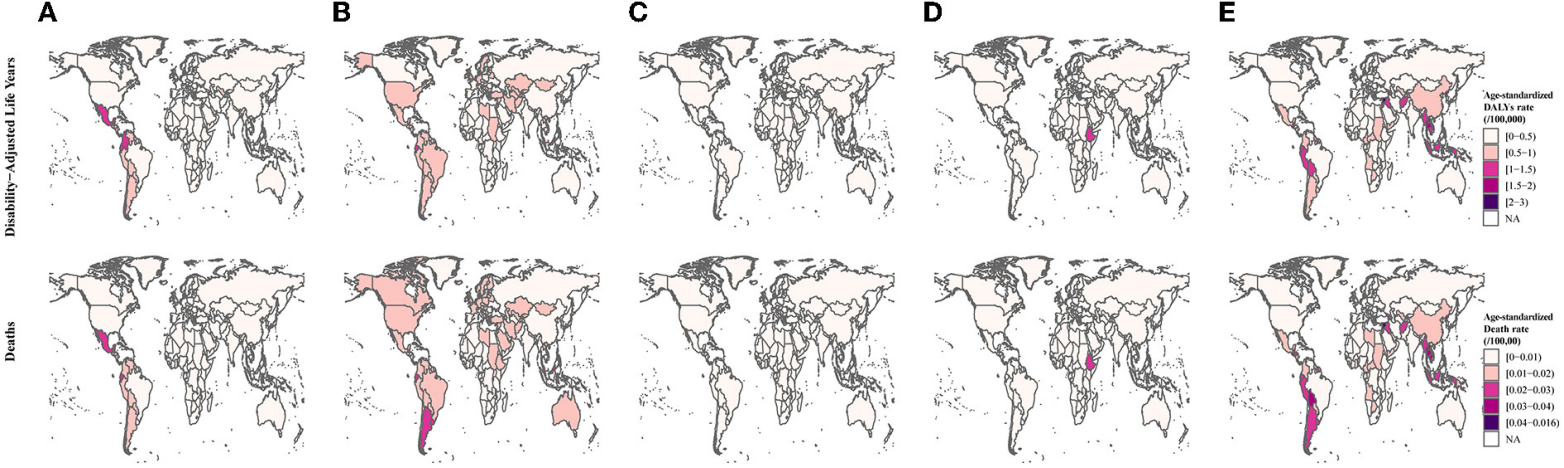
Differences in types of leukemia are attributable to occupational risk. **(A)** Acute lymphoid leukemia. **(B)** Acute myeloid leukemia. **(C)** Chronic lymphoid leukemia. **(D)** Chronic myeloid leukemia. **(E)** Other leukemia.

**Figure 3:**
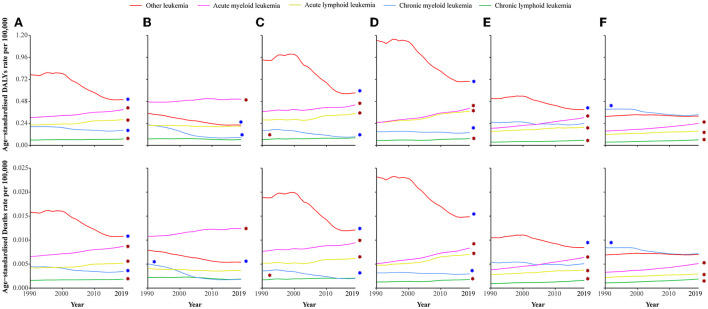
Age-standardized DALY and death rates of different types of leukemia attributable to occupational risk among SDI quintiles between 1990 and 2019. **(A)** Age-standardized DALY and death rates of leukemia are attributable to occupational risk globally. **(B)** High-SDI countries. **(C)** Higher-middle-SDI countries. **(D)** Middle-SDI countries. **(E)** Lower-middle-SDI countries. **(F)** Low-SDI countries. SDI, social development index. The red asterisk represents a significant rise trend, and the blue asterisk represents a significant decrease trend.

In 2019, ALL and AML were the leading causes of leukemia attributable to occupational risk-related DALYs and death rates. Both ALL and AML attributable to occupational risk were heavy in Central Latin America, Andean Latin America, and Southern Latin America (Figure 2, Supplementary Table 1). Globally, the age-standardized DALYs and death rates of ALL, AML, and CLL were presented a significantly increase trends with EAPCs [0.78% (95% CI: 0.65–0.91%), 0.87% (95% CI: 0.81–0.93%), and 0.66% (95% CI: 0.51–0.81%) for DALYs, respectively, and 0.75% (95% CI: 0.68–0.82%), 0.96% (95% CI: 0.91–1.01%), and 0.55% (95% CI: 0.43–0.68%) for death, respectively], whereas the age-standardized DALYs and death rates of CML and other leukemia were significantly decreased. For SDI quintiles, except for the high SDI level region, the ALL, AML, and CLL were significantly increased in other SDI levels region (Figure 3, Supplementary Table 2).

### Leukemia attributable to occupational risk age distribution structure

**Figure 4:**
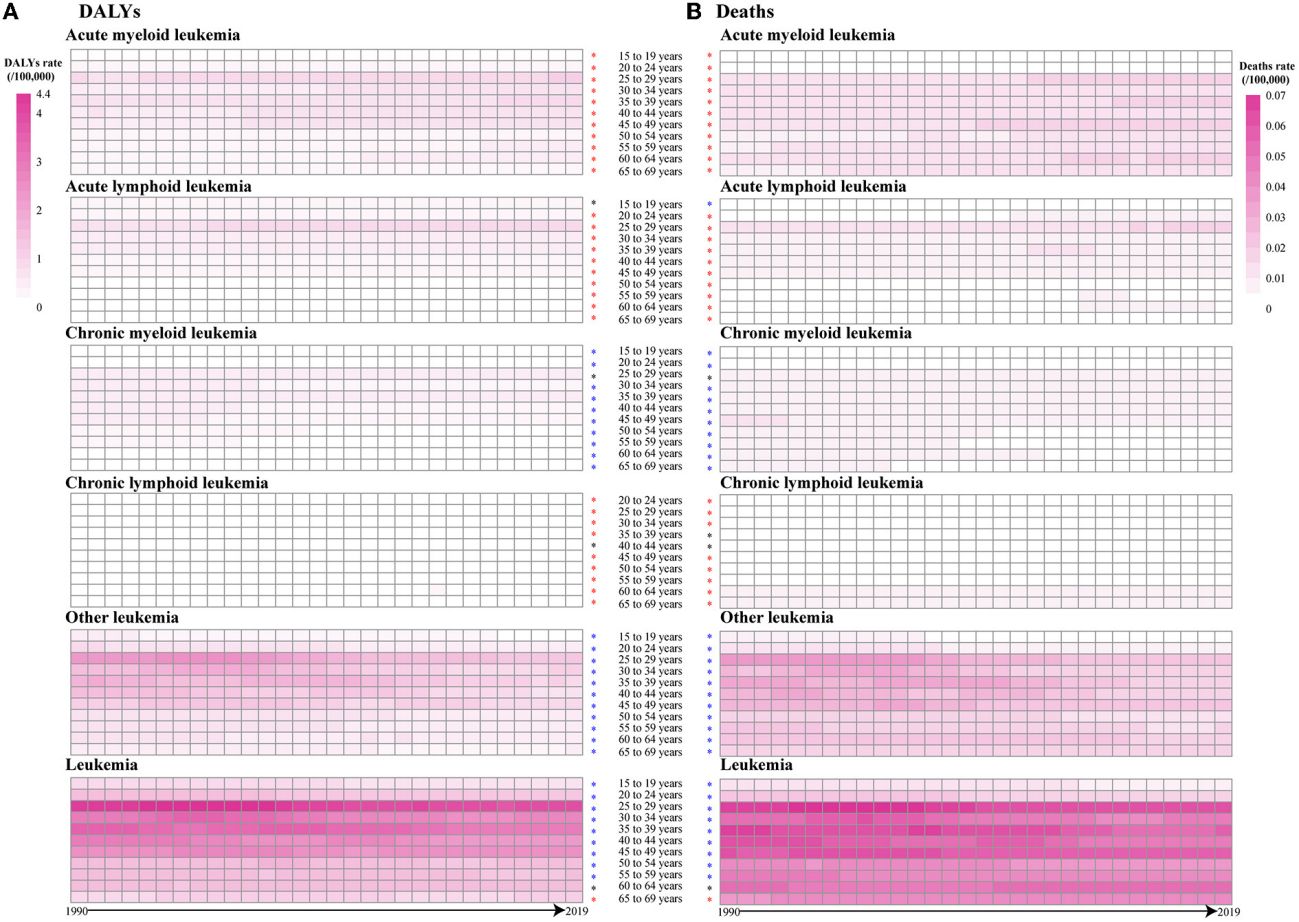
The DALY and death rates of leukemia were attributable to occupational risk among different age groups between 1990 and 2019. Red asterisks represent an uptrend, blue asterisks represent a decline, and black asterisks represent a steady trend. **(A)** DALYs and **(B)** Deaths.

We analyzed the DALYs and death rates of leukemia attributable to occupational risk in three different age groups. The results indicated that most DALYs and deaths occurred in 25–29 years in the globe. Overall, the leukemia attributable to occupational risk was significantly increased in people aged 65–69 years [0.42% (95% CI: 0.30–0.55%) for DALYs and 0.38% (95% CI: 0.26–0.51%) for death]. The burdens of ALL, AML, and CLL were increased in almost all age groups while decreased in CML and other leukemia (Figure 4).

### Relationship between SDI and the burdens of leukemia attributable to occupational risk

**Figure 5:**
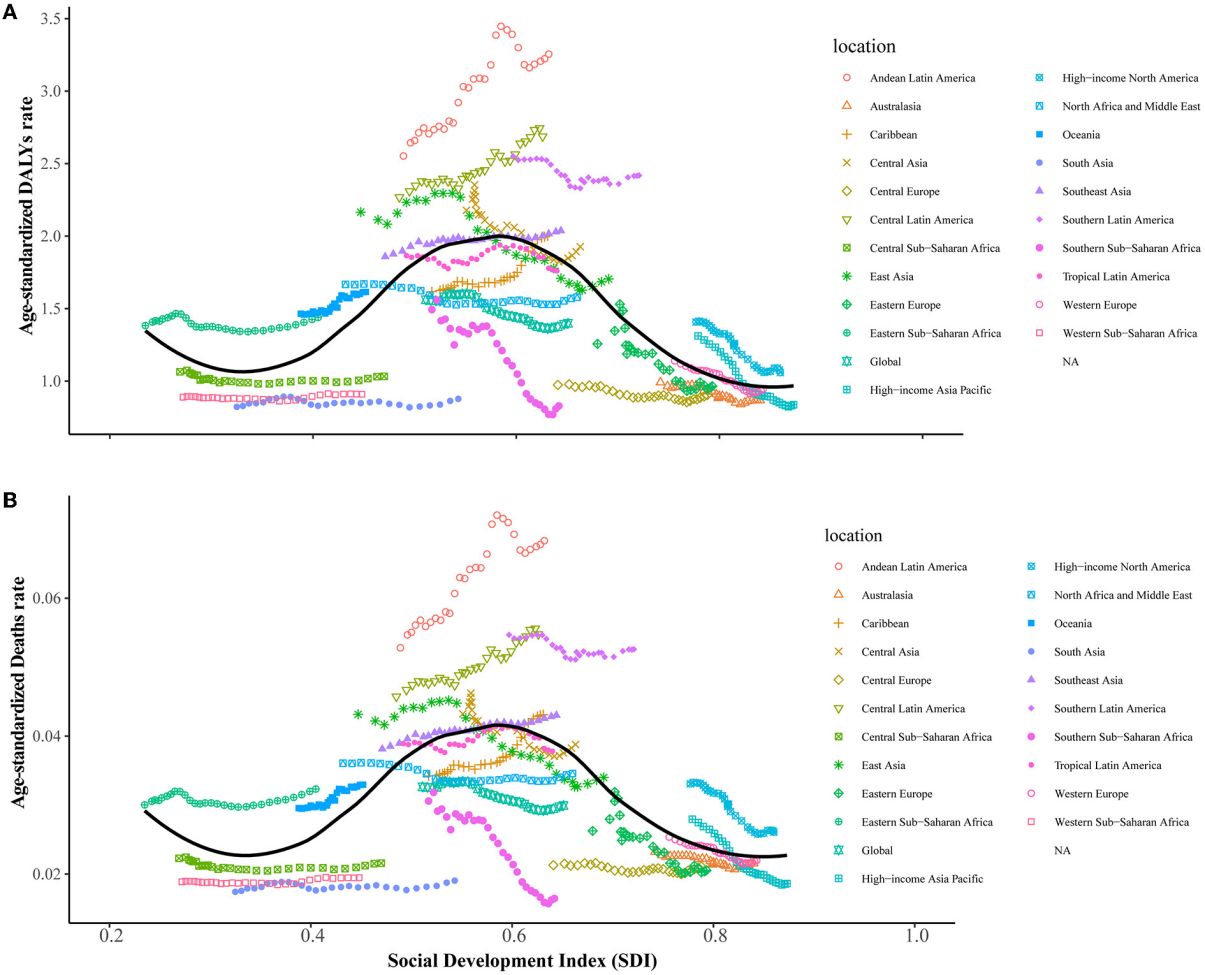
Age-standardized DALY and death rates were attributable to leukemia attributable to occupational risk across 21 GBD regions by the socio-demographic index for both sexes combined in 1990–2019. **(A)** Age-standardized DALYs rates. **(B)** Age-standardized death rates.

In 2019, the highest age-standardized rates of leukemia attributable to occupational risk-related deaths and DALYs were observed in countries in the Middle-SDI [1.6778 (95% UI: 0.7894–2.4881) DALYs per 100,000 people and 0.0348 (95% UI: 0.0164–0.052) deaths per 100,000 people]. Figure 5 and Supplementary Figure 1 show the changes in age-standardized DALYs and death rates across the SDI by region from 1990 to 2019. Five regions with the highest SDI exhibited a decline in the age-standardized rate of leukemia attributable to occupational risk-related DALYs and deaths, whereas five regions with the lowest SDI experienced an increasing trend. The regions with middle SDI show greatly varied. The associations between age-standardized DALYs and death rates and SDI across countries in 2019 are shown in Supplementary Figure 1.

## Discussion

In this study, we reported the disease burden of leukemia attributable to occupational risk-related deaths and their trends from 1990 to 2019 at the global, regional, and country levels. Our findings showed several key points. Changes between different countries and regions in the burden and trends in total and particular leukemia attributable to occupational risk-related deaths across the globe were found in our research. In general, regions of Andean Latin America, Central Latin America, and the Caribbean were the so-called hotspot regions with the highest age-standardized rates of total leukemia attributable to occupational risk-related deaths in 2019. Overall, most countries and regions showed a decrease in age-standardized DALYs and death rates. The DALYs and deaths of leukemia caused by occupational risk in elderly people is higher than that in other age groups, and the burdens are on the rise.

We analyzed the epidemiological trends of leukemia attributable to occupational risk by calculating the EAPC values from 1990 to 2019. As everyone knows, aging is an important factor contributing to leukemogenesis. Accompanied by aging gene mutations, changes in internal environmental homeostasis and mitochondrial dysfunction make the risk of leukemia higher in the elderly than in the young people (32–35). The marked increase in leukemia attributable to occupational risk in Latin America in terms of deaths and DALYs can be partially explained by the fact that humans in these countries and regions are mostly engaged in physical labor and have more opportunities to come into contact with toxic substances such as formaldehyde and benzene. Besides, long-term poverty, poor access to healthcare, a lower awareness regarding occupational risk, inadequate prevention measures and screening, and a lower proportion of treatment together make low-income countries more susceptible to leukemia attributable to occupational risk than high-income countries (36–38). Therefore, in the subsequent campaign to eliminate leukemia attributable to occupational risk, more attention and health resources were warranted in developing countries and low-income regions.

Of note, our research found that, among all leukemia caused by occupational risk, the age-standardized DALYs and death rates were particularly observed in ALL and AML. The possible reasons can be listed as follows. First, the standard of leukemia classification has changed. For example, the World Health Organization (WHO) classification (2, 39), recognized as a standard for disease diagnosis and public health monitoring worldwide, has been revised from the primary to the 5^th^ edition during the past 60 years (2). French-American-British (FAB) classification (40) and MICM classification (Morphology, Immunology, Cytogenetics, and Molecular) classification (41). Meanwhile, the diagnostic criteria for leukemia have also changed (42, 43). In terms of the countries and regions' distribution of these two kinds of leukemia, in Mexico and Latin America, the age-standardized DALYs and death rates were the highest, possibly because of the most common of these two types of leukemia in young adults and regional economic development. Thus, accurate surveillance data were important for developing a prevention-and-control program and providing valuable countermeasures to estimate the impact of those programs (44, 45). Elderly people were found to have disproportionately high DALYs and death rates in total leukemia attributable to occupational risk-related deaths, which might be due to age factors. For one thing, the DALYs and death rates of leukemia will increase with age, so the DALYs and death rates of the elderly will be higher than that of young adults. For another, if the elderly were exposed to toxic and harmful substances such as formaldehyde and benzene during adolescence, these substances will not immediately cause reactions after exposure but will slowly accumulate in the body, and symptoms will slowly emerge with age (46, 47). Our findings call for an urgent need to accelerate efforts to reduce leukemia attributable to the occupational risk burden in elderly people. Male individuals generally had higher age-standardized DALYs and death rates than female individuals for leukemia attributable to occupational risk-related deaths, possibly because men were the main part of social labor and were more engaged in heavy physical labor, thus, they had more chances to contact toxic and harmful substances. In addition, the burden of leukemia itself is higher in men than in women (48, 49).

Most countries had a decrease in age-standardized rates of DALYs and death rates for leukemia attributable to occupational risk-related deaths, whereas the absolute DALYs and death cases increased from 1990 to 2019. Leukemia attributable to occupational risk-related deaths still represents a global public health challenge, especially in Latin America and other developing countries, where more attention and health prevention services are warranted. Our study also suggested an upward trend of leukemia attributable to occupation risk among elderly people. ALL, AML, and CLL showed an upward trend in almost all age groups. Thus, there remains an urgent need to accelerate efforts to reduce leukemia attributable to occupational risk-related death burden in this population and specific causes.

## Data availability statement

The original contributions presented in the study are included in the article/Supplementary material, further inquiries can be directed to the corresponding author/s.

## Author contributions

WX designed the study. DX, CC, HS, YY, and YX accessed and verified the data. XY, HT, AL, and JJ analyzed the data and interpreted the results. YS, YH, XT, and DC wrote the manuscript. All authors revised the manuscript from the preliminary draft to submission.

## Funding

This study was funded by the National Natural Science Foundation of China (81871709).

## Conflict of interest

The authors declare that the research was conducted in the absence of any commercial or financial relationships that could be construed as a potential conflict of interest.

## Publisher's note

All claims expressed in this article are solely those of the authors and do not necessarily represent those of their affiliated organizations, or those of the publisher, the editors and the reviewers. Any product that may be evaluated in this article, or claim that may be made by its manufacturer, is not guaranteed or endorsed by the publisher.

## Supplementary material

Supplementary Figure 1 Age-standardized DALY and death rates were attributable to leukemia attributable to occupational risk across countries and territories by the socio-demographic index for both sexes combined in 2019. **(A)** Age-standardized DALYs rates. **(B)** Age-standardized death rates.

Supplementary Table 1 Differences in types of leukemia attributable to occupational risk in 21 GBD regions.

Supplementary Table 2 The trends of different types of leukemia attributable to occupational risk among SDI quintiles between 1990 and 2019.

## References

[1] Li J Su H Chen H Futscher BW. Optimal search-based gene subset selection for gene array cancer classification. IEEE Trans Inf Technol Biomed. (2007) 11:398–405. 10.1109/TITB.2007.892693 17674622

[2] Khoury JD Solary E Abla O Akkari Y Alaggio R Apperley JF. The 5th edition of the world health organization classification of haematolymphoid tumours: myeloid and histiocytic/dendritic neoplasms. Leukemia. (2022) 36:1703–19. 10.1038/s41375-022-01613-1 35732831 PMC9252913

[3] Pathinarupothi RK Durga P Rangan ES. Data to diagnosis in global health: a 3P approach. BMC Med Inform Decis Mak. (2018) 18:78. 10.1186/s12911-018-0658-y 30180839 PMC6124014

[4] Schwalbe EC Lindsey JC Nakjang S Crosier S Smith AJ Hicks D. Novel molecular subgroups for clinical classification and outcome prediction in childhood medulloblastoma: a cohort study. Lancet Oncol. (2017) 18:958–71. 10.1016/S1470-2045(17)30243-7 28545823 PMC5489698

[5] Duncavage EJ Schroeder MC O'Laughlin M Wilson R MacMillan S Bohannon A. Genome sequencing as an alternative to cytogenetic analysis in myeloid cancers. N Engl J Med. (2021) 384:924–35. 10.1056/NEJMoa2024534 33704937 PMC8130455

[6] Siegel RL Miller KD Jemal A. Cancer statistics, 2019. CA Cancer J Clin. (2019) 69:7–34. 10.3322/caac.21551 30620402

[7] Song X Peng Y Wang X Chen Y Jin L Yang T. Incidence, survival, and risk factors for adults with acute myeloid leukemia not otherwise specified and acute myeloid leukemia with recurrent genetic abnormalities: analysis of the surveillance, epidemiology, and end results (SEER) database, 2001-2013. Acta Haematol. (2018) 139:115–27. 10.1159/000486228 29455198

[8] Qiu KY Liao XY Liu Y Huang K Li Y Fang JP. Poor outcome of pediatric patients with acute myeloid leukemia harboring high FLT3/ITD allelic ratios. Nat Commun. (2022) 13:3679. 10.1038/s41467-022-31489-9 35760968 PMC9237020

[9] Sliwa-Tytko P Kaczmarska A Lejman M Zawitkowska J. Neurotoxicity associated with treatment of acute lymphoblastic leukemia chemotherapy and immunotherapy. Int J Mol Sci. (2022) 23:5515. 10.3390/ijms23105515 35628334 PMC9146746

[10] Prabhakar SK Ryu S Jeong IC Won DO. A dual level analysis with evolutionary computing and swarm models for classification of leukemia. Biomed Res Int. (2022) 2022:2052061. 10.1155/2022/2052061 35663047 PMC9162867

[11] Varghese JV Sebastian EM Iqbal T Tom AA. Pesticide applicators and cancer: a systematic review. Rev Environ Health. (2021) 36:467–76. 10.1515/reveh-2020-0121 34821114

[12] Proceedings of the IARC Working Group on the Evaluation of Carcinogenic Risks to Humans. Epstein-Barr virus and kaposi's sarcoma herpesvirus/human herpesvirus 8. Lyon, France, 17-24 June 1997. IARC Monogr Eval Carcinog Risks Hum. (1997) 70:1–492.9705682

[13] Zheng Y Yu Q Lin Y Zhou Y Lan L Yang S. Global burden and trends of sexually transmitted infections from 1990 to 2019: an observational trend study. Lancet Infect Dis. (2022) 22:541–51. 10.1016/S1473-3099(21)00448-5 34942091

[14] Driscoll TR Carey RN Peters S Glass DC Benke G Reid A. The Australian work exposures study: prevalence of occupational exposure to formaldehyde. Ann Occup Hyg. (2016) 60:132–8. 10.1093/annhyg/mev058 26342091

[15] Vecchio D Sasco AJ Cann CI. Occupational risk in health care and research. Am J Ind Med. (2003) 43:369–97. 10.1002/ajim.10191 12645094

[16] Checkoway H Boffetta P Mundt DJ Mundt KA. Critical review and synthesis of the epidemiologic evidence on formaldehyde exposure and risk of leukemia and other lymphohematopoietic malignancies. Cancer Causes Control. (2012) 23:1747–66. 10.1007/s10552-012-0055-2 22983399 PMC3465649

[17] IARC Working Group on the Evaluation of Carcinogenic Risks to Humans. Chemical agents and related occupations. IARC Monogr Eval Carcinog Risks Hum. (2012) 100:9–562. 23189753 PMC4781612

[18] Baan R Grosse Y Straif K Secretan B El Ghissassi F Bouvard V. A review of human carcinogens—part F: chemical agents and related occupations. Lancet Oncol. (2009) 10:1143–4. 10.1016/S1470-2045(09)70358-4 19998521

[19] Collins JJ Lineker GA. A review and meta-analysis of formaldehyde exposure and leukemia. Regul Toxicol Pharmacol. (2004) 40:81–91. 10.1016/j.yrtph.2004.04.006 15450712

[20] Zhang L Steinmaus C Eastmond DA Xin XK Smith MT. Formaldehyde exposure and leukemia: a new meta-analysis and potential mechanisms. Mutat Res. (2009) 681:150–68. 10.1016/j.mrrev.2008.07.002 18674636

[21] Blue I Harpham T. The world bank world development report 1993: investing in health. Reveals the burden of common mental disorders, but ignores its implications. Br J Psychiatry. (1994) 165:9–12. 10.1192/bjp.165.1.9 7953063

[22] GBD Tuberculosis Collaborators. Global regional, and national burden of tuberculosis, 1990-2016: results from the global burden of diseases, injuries, and risk factors 2016 study. Lancet Infect Dis. (2018) 18:1329–49. 10.1016/S1473-3099(18)30625-X 30507459 PMC6250050

[23] GBD 2019 Diseases and Injuries Collaborators. Global burden of 369 diseases and injuries in 204 countries and territories, 1990-2019: a systematic analysis for the global burden of disease study 2019. Lancet. (2020) 396:1204–22. 10.1016/S0140-6736(20)30925-9 33069326 PMC7567026

[24] Wang H Zhao S Wang S Zheng Y Wang S Chen H. Global magnitude of encephalitis burden and its evolving pattern over the past 30 years. J Infect. (2022) 84:777–87. 10.1016/j.jinf.2022.04.026 35452715

[25] Zhao S Wang H Chen H Wang S Ma J Zhang D. Global magnitude and long-term trend of ischemic heart disease burden attributed to household air pollution from solid fuels in 204 countries and territories, 1990-2019. Indoor Air. (2022) 32:e12981. 10.1111/ina.12981 35037299

[26] Landrigan PJ Fuller R Acosta NJR Adeyi O Arnold R Basu NN. The lancet commission on pollution and health. Lancet. (2018) 391:462–512. 10.1016/S0140-6736(17)32345-0 29056410

[27] Vanka KS Shukla S Gomez HM James C Palanisami T Williams K. Understanding the pathogenesis of occupational coal and silica dust-associated lung disease. Eur Respir Rev. (2022) 31:210250. 10.1183/16000617.0250-2021 35831008 PMC9724915

[28] GBD 2017 Risk Factor Collaborators. Global, regional, and national comparative risk assessment of 84 behavioural, environmental and occupational, and metabolic risks or clusters of risks for 195 countries and territories, 1990-2017: a systematic analysis for the global burden of disease study 2017. Lancet. (2018) 392:1923–94. 10.1016/S0140-6736(18)32225-6 30496105 PMC6227755

[29] GBD 2017 Causes of Death Collaborators. Global, regional, and national age-sex-specific mortality for 282 causes of death in 195 countries and territories, 1980-2017: a systematic analysis for the Global Burden of Disease Study 2017. Lancet. (2018) 392:1736–88. 10.1016/S0140-6736(18)32203-7 30496103 PMC6227606

[30] Xie L Shang ZJ. Oral cancer incidence, mortality, and mortality-to-incidence ratio are associated with human development index in China, 1990-2019. Biomed Res Int. (2022) 2022:6457840. 10.1155/2022/6457840 35800221 PMC9256441

[31] Cao G Liu J Liu M. Global, regional, and national incidence and mortality of neonatal preterm birth, 1990-2019. JAMA Pediatr. (2022) 176:787–96. 10.1001/jamapediatrics.2022.1622 35639401 PMC9157382

[32] Zjablovskaja P Florian MC. Acute myeloid leukemia: aging and epigenetics. Cancers. (2019) 12:103. 10.3390/cancers12010103 31906064 PMC7017261

[33] Kerk S Lin L Myers AL Sutton DJ Andren A Sajjakulnukit P. Metabolic requirement for GOT2 in pancreatic cancer depends on environmental context. Elife. (2022) 11:e73245. 10.7554/eLife.73245 35815941 PMC9328765

[34] Wong CC Wu JL Ji F Kang W Bian X Chen H. The cholesterol uptake regulator PCSK9 promotes and is a therapeutic target in APC/KRAS-mutant colorectal cancer. Nat Commun. (2022) 13:3971. 10.1038/s41467-022-31663-z 35803966 PMC9270407

[35] Gao M Huang X Wu Z Wang L Yuan S Du Z. Synthesis of a versatile mitochondria-targeting small molecule for cancer near-infrared fluorescent imaging and radio/photodynamic/photothermal synergistic therapies. Mater Today Bio. (2022) 15:100316. 10.1016/j.mtbio.2022.100316 35721281 PMC9198388

[36] Aral SO Fenton KA Holmes KK. Sexually transmitted diseases in the USA: temporal trends. Sex Transm Infect. (2007) 83:257–66. 10.1136/sti.2007.026245 17664359 PMC2598671

[37] Trani JF Moodley J Maw MTT Babulal GM. Association of multidimensional poverty with dementia in adults aged 50 years or older in South Africa. JAMA Netw Open. (2022) 5:e224160. 10.1001/jamanetworkopen.2022.4160 35333360 PMC8956981

[38] Rasella D Alves FJO Reboucas P de Jesus GS Barreto ML Campello T. Long-term impact of a conditional cash transfer programme on maternal mortality: a nationwide analysis of Brazilian longitudinal data. BMC Med. (2021) 19:127. 10.1186/s12916-021-01994-7 34059069 PMC8166529

[39] Alaggio R Amador C Anagnostopoulos I Attygalle AD Araujo IBO Berti E. The 5th edition of the world health organization classification of haematolymphoid tumours: lymphoid neoplasms. Leukemia. (2022) 36:1720–48. 10.1038/s41375-022-01620-2 35732829 PMC9214472

[40] Paolillo R Boulanger M Gatel P Gabellier L De Toledo M Tempe D. The NADPH oxidase NOX2 is a marker of adverse prognosis involved in chemoresistance of acute myeloid leukemias. Haematologica. (2022) 107:2562–75. 10.3324/haematol.2021.279889 35172562 PMC9614539

[41] Feng L Li Y Li Y Jiang Y Wang N Yuan D. Whole exome sequencing detects CHST3 mutation in patient with acute promyelocytic leukemia: a case report. Medicine. (2018) 97:e12214. 10.1097/MD.0000000000012214 30200136 PMC6133617

[42] Mrozek K. Molecular cytogenetics in acute myeloid leukemia in adult patients: practical implications. Pol Arch Intern Med. (2022) 132:16300. 10.20452/pamw.16300 35848612

[43] Kulp M Siemund AL Larghero P Dietz A Alten J Cario G. The immune checkpoint ICOSLG is a relapse-predicting biomarker and therapeutic target in infant t(4;11) acute lymphoblastic leukemia. iScience. (2022) 25:104613. 10.1016/j.isci.2022.104613 35800767 PMC9253708

[44] Wang L Wang Y Jin S Wu Z Chin DP Koplan JP. Emergence and control of infectious diseases in China. Lancet. (2008) 372:1598–605. 10.1016/S0140-6736(08)61365-3 18930534 PMC7138027

[45] Amato E Riess M Thomas-Lopez D Linkevicius M Pitkanen T Wolkowicz T. Epidemiological and microbiological investigation of a large increase in vibriosis, northern Europe, 2018. Euro Surveill. (2022) 27:2101088. 10.2807/1560-7917.ES.2022.27.28.2101088 35837965 PMC9284918

[46] Protano C Buomprisco G Cammalleri V Pocino RN Marotta D Simonazzi S. The carcinogenic effects of formaldehyde occupational exposure: a systematic review. Cancers. (2021) 14:165. 10.3390/cancers14010165 35008329 PMC8749969

[47] Mozzoni P Pinelli S Corradi M Ranzieri S Cavallo D Poli D. Environmental/Occupational exposure to radon and non-pulmonary neoplasm risk: a review of epidemiologic evidence. Int J Environ Res Public Health. (2021) 18:10466. 10.3390/ijerph181910466 34639764 PMC8508162

[48] Ren HM Liao MQ Tan SX Cheng C Zhu S Zheng L. Global, regional, and national burden of cancer in children younger than 5 years, 1990-2019: analysis of the global burden of disease study 2019. Front Public Health. (2022) 10:910641. 10.3389/fpubh.2022.910641 35801252 PMC9255714

[49] Khorrami Z Pourkhosravani M Eslahi M Rezapour M Akbari ME Amini H. Multiple air pollutants exposure and leukaemia incidence in Tehran, Iran from 2010 to 2016: a retrospective cohort study. BMJ Open. (2022) 12:e060562. 10.1136/bmjopen-2021-060562 35732402 PMC9226961

