# Supplementary figures and images for: Global disease burden and trends of leukemia attributable to occupational risk from 1990 to 2019: An observational trend study
Source: Front Public Health. 2022 Nov 14;10:1015861. doi: 10.3389/fpubh.2022.1015861 (PMC9703980; doi:10.3389/fpubh.2022.1015861)

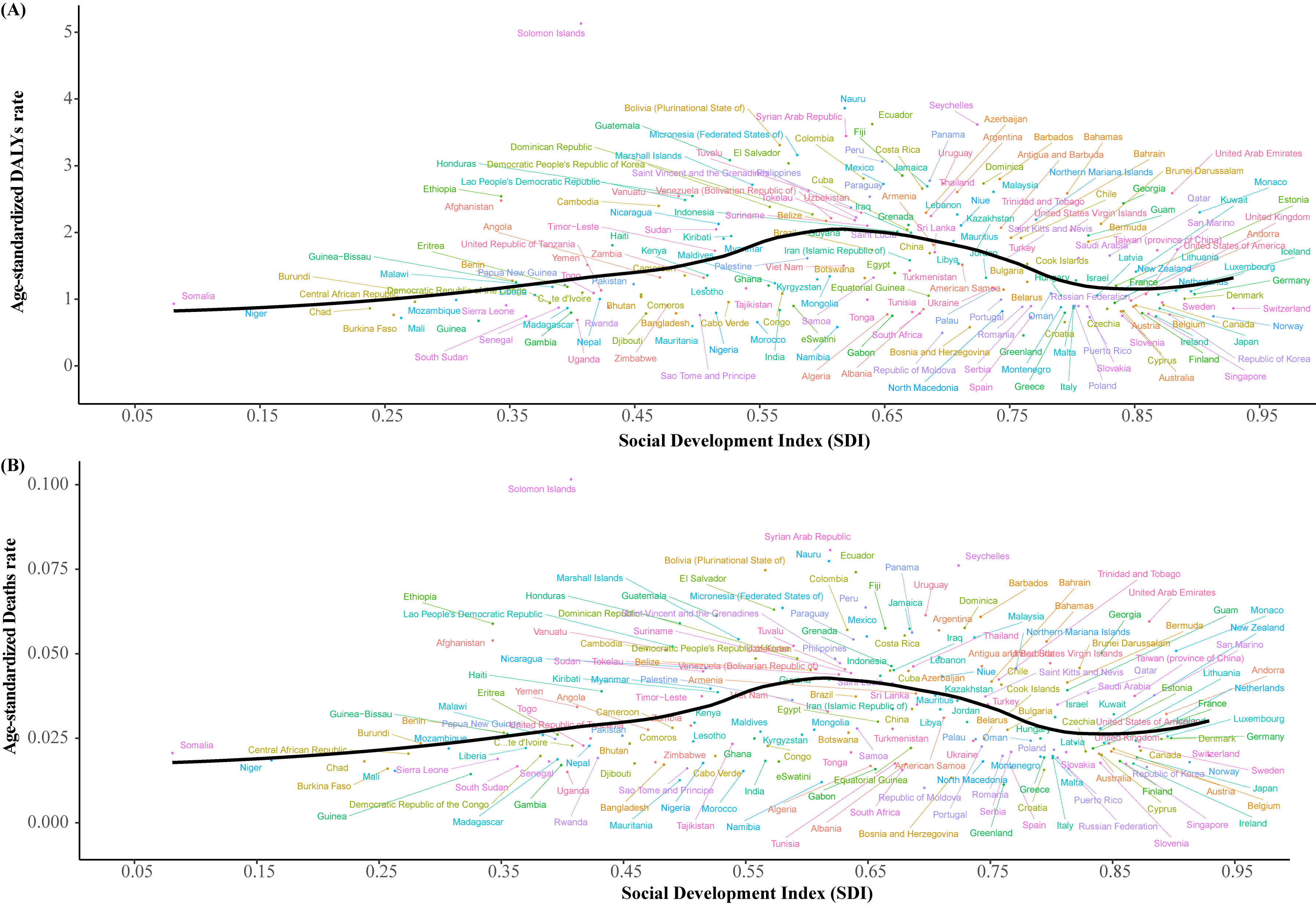

Supplement: Supplementary Figure 1 — Age-standardized DALY and death rates were attributable to leukemia attributable to occupational risk across countries and territories by the socio-demographic index for both sexes combined in 2019. (A) Age-standardized DALYs rates. (B) Age-standardized death rates. [file Image_1.tif]
