# Supplementary material for: Global disease burden and trends of leukemia attributable to occupational risk from 1990 to 2019: An observational trend study
Source: Front Public Health. 2022 Nov 14;10:1015861. doi: 10.3389/fpubh.2022.1015861 (PMC9703980; doi:10.3389/fpubh.2022.1015861)
Supplement: Supplementary Table 1 — Differences in types of leukemia attributable to occupational risk in 21 GBD regions. [file Table_1.docx]

**Table S 1.** Differences types of leukemia attributable to occupational risk in 21 GDB regions

|  | **Acute lymphoid leukemia** | | | | **Acute myeloid leukemia** | | | | **Chronic lymphoid leukemia** | | | | **Chronic myeloid leukemia** | | | | **Other leukemia** | | | |
| --- | --- | --- | --- | --- | --- | --- | --- | --- | --- | --- | --- | --- | --- | --- | --- | --- | --- | --- | --- | --- |
| **Location name** | **DALYs** **in 2019** | **EAPC** | **Deaths in 2019** | **EAPC** | **DALYs in 2019** | **EAPC** | **Deaths in 2019** | **EAPC** | **DALYs in 2019** | **EAPC** | **Deaths in 2019** | **EAPC** | **DALYs in 2019** | **EAPC** | **Deaths in 2019** | **EAPC** | **DALYs in 2019** | **EAPC** | **Deaths in 2019** | **EAPC** |
| Andean Latin America | 0.8612 | 1.87% (1.59% - 2.16%)* | 0.0163 | 1.9% (1.66% - 2.14%)* | 0.8337 | 2.49% (2.36% - 2.62%)* | 0.018 | 2.53% (2.4% - 2.66%)* | 0.0831 | 1.77% (0.71% - 2.84%)* | 0.0021 | 1.86% (0.75% - 2.98%)* | 0.2079 | 0.59% (-0.12% - 1.3%) | 0.0046 | 0.67% (0% - 1.34%)* | 1.2693 | -0.45% (-1.44% - 0.54%) | 0.0272 | -0.34% (-1.29% - 0.62%) |
| Australasia | 0.1596 | -1.02% (-1.33% - -0.7%)* | 0.003 | -0.99% (-1.12% - -0.86%)* | 0.4973 | 0.28% (0.09% - 0.47%)* | 0.0128 | 0.56% (0.32% - 0.8%)* | 0.0659 | 0.15% (-0.04% - 0.34%) | 0.0021 | 0.01% (-0.22% - 0.25%) | 0.0639 | -3.58% (-3.9% - -3.26%)* | 0.0015 | -3.68% (-3.99% - -3.36%)* | 0.0806 | 0.84% (-0.11% - 1.79%) | 0.0022 | 0.95% (0.64% - 1.27%)* |
| Caribbean | 0.2756 | 0.55% (0.4% - 0.71%)* | 0.0053 | 0.66% (0.51% - 0.82%)* | 0.6177 | 1.23% (1.02% - 1.43%)* | 0.0132 | 1.34% (1.15% - 1.52%)* | 0.0687 | 1.02% (0.71% - 1.33%)* | 0.0021 | 1.14% (0.82% - 1.47%)* | 0.2136 | -0.58% (-0.92% - -0.23%)* | 0.0049 | -0.41% (-0.79% - -0.04%)* | 0.8261 | 0.92% (0.49% - 1.35%)* | 0.0176 | 0.91% (0.52% - 1.3%)* |
| Central Asia | 0.3924 | -0.07% (-0.22% - 0.08%) | 0.0074 | -0.06% (-0.21% - 0.1%) | 0.6965 | 0.62% (0.36% - 0.88%)* | 0.014 | 0.67% (0.43% - 0.9%)* | 0.0891 | -0.11% (-0.5% - 0.28%) | 0.0022 | 0.07% (-0.32% - 0.46%) | 0.1353 | -1.21% (-1.82% - -0.6%)* | 0.0029 | -1.15% (-1.63% - -0.67%)* | 0.6136 | -1.35% (-1.64% - -1.05%)* | 0.0123 | -1.27% (-1.57% - -0.97%)* |
| Central Europe | 0.1602 | -0.48% (-0.65% - -0.31%)* | 0.003 | -0.62% (-0.77% - -0.47%)* | 0.4147 | 0.78% (0.64% - 0.92%)* | 0.0096 | 1.06% (0.94% - 1.18%)* | 0.096 | 2.04% (1.87% - 2.2%)* | 0.0029 | 2.24% (2.07% - 2.4%)* | 0.0573 | -2.96% (-3.48% - -2.43%)* | 0.0013 | -2.78% (-3.25% - -2.31%)* | 0.1748 | -1.51% (-1.8% - -1.2%)* | 0.0043 | -1.25% (-1.53% - -0.98%)* |
| Central Latin America | 1.0112 | 1.85% (1.51% - 2.19%)* | 0.019 | 1.87% (1.64% - 2.1%)* | 0.7998 | 1.58% (1.2% - 1.96%)* | 0.0167 | 1.69% (1.37% - 2%)* | 0.0556 | 0.7% (0.38% - 1.03%)* | 0.0016 | 0.77% (0.49% - 1.05%)* | 0.1834 | -1.04% (-1.55% - -0.52%)* | 0.0041 | -0.94% (-1.38% - -0.51%)* | 0.6378 | -1.06% (-1.3% - -0.82%)* | 0.0134 | -0.97% (-1.19% - -0.74%)* |
| Central Sub-Saharan Africa | 0.1012 | 0.75% (0.6% - 0.89%)* | 0.0018 | 0.66% (0.51% - 0.8%)* | 0.1748 | 0.73% (0.58% - 0.89%)* | 0.0035 | 0.68% (0.53% - 0.83%)* | 0.0741 | 2.81% (2.58% - 3.04%)* | 0.0021 | 2.85% (2.59% - 3.11%)* | 0.1867 | 0.01% (-0.31% - 0.33%) | 0.0039 | -0.07% (-0.36% - 0.22%) | 0.4958 | -0.79% (-0.95% - -0.64%)* | 0.0102 | -0.86% (-1% - -0.71%)* |
| East Asia | 0.3894 | 2.14% (1.86% - 2.42%)* | 0.0071 | 1.92% (1.69% - 2.15%)* | 0.2336 | 1.68% (1.53% - 1.84%)* | 0.0048 | 1.73% (1.61% - 1.85%)* | 0.1066 | 2.07% (1.6% - 2.54%)* | 0.0022 | 1.98% (1.55% - 2.42%)* | 0.0352 | -1.58% (-2.12% - -1.03%)* | 0.0007 | -1.69% (-2.13% - -1.25%)* | 0.9395 | -1.99% (-2.4% - -1.58%)* | 0.0192 | -1.9% (-2.1% - -1.7%)* |
| Eastern Europe | 0.2821 | -0.69% (-1.04% - -0.33%)* | 0.0053 | -0.69% (-1.19% - -0.19%)* | 0.3193 | -0.93% (-1.55% - -0.31%)* | 0.0067 | -0.83% (-1.43% - -0.23%)* | 0.0867 | 0.08% (-0.54% - 0.71%) | 0.0024 | 0.15% (-0.46% - 0.77%) | 0.1092 | -0.82% (-1.54% - -0.09%)* | 0.0024 | -0.76% (-1.47% - -0.04%)* | 0.1677 | -1.34% (-2.31% - -0.36%)* | 0.0037 | -1.27% (-2.12% - -0.41%)* |
| Eastern Sub-Saharan Africa | 0.209 | 1.2% (1.04% - 1.36%)* | 0.004 | 1.19% (1.05% - 1.34%)* | 0.2769 | 1.8% (1.58% - 2.01%)* | 0.006 | 1.87% (1.72% - 2.02%)* | 0.1023 | 2.13% (1.9% - 2.36%)* | 0.0034 | 2.34% (2.12% - 2.57%)* | 0.5213 | -0.89% (-0.99% - -0.79%)* | 0.0115 | -0.8% (-0.89% - -0.71%)* | 0.3294 | -0.15% (-0.29% - -0.01%)* | 0.0073 | -0.05% (-0.19% - 0.1%) |
| High-income Asia Pacific | 0.2088 | -0.4% (-0.61% - -0.19%)* | 0.0034 | -0.82% (-1.02% - -0.61%)* | 0.4164 | -0.45% (-0.56% - -0.34%)* | 0.0101 | -0.11% (-0.21% - 0%)* | 0.0134 | 0.28% (0% - 0.56%)* | 0.0003 | -0.08% (-0.36% - 0.2%) | 0.0523 | -5.05% (-5.37% - -4.73%)* | 0.0011 | -5.15% (-5.46% - -4.84%)* | 0.1465 | -3.37% (-3.7% - -3.04%)* | 0.0038 | -2.82% (-3.06% - -2.57%)* |
| High-income North America | 0.1969 | -0.43% (-0.63% - -0.23%)* | 0.0038 | -0.38% (-0.56% - -0.21%)* | 0.5149 | -0.07% (-0.27% - 0.13%) | 0.0131 | 0.24% (0.05% - 0.43%)* | 0.0704 | -1.07% (-1.23% - -0.9%)* | 0.0022 | -1.09% (-1.24% - -0.94%)* | 0.064 | -4.58% (-5.08% - -4.08%)* | 0.0015 | -4.49% (-4.98% - -4%)* | 0.2127 | -1.62% (-1.98% - -1.25%)* | 0.0055 | -1.39% (-1.54% - -1.25%)* |
| North Africa and Middle East | 0.2721 | -0.02% (-0.18% - 0.14%) | 0.0052 | 0% (-0.15% - 0.15%) | 0.5308 | 0.57% (0.48% - 0.66%)* | 0.0114 | 0.64% (0.54% - 0.74%)* | 0.0573 | 0.6% (0.39% - 0.81%)* | 0.0016 | 0.67% (0.44% - 0.9%)* | 0.2102 | -0.95% (-1.09% - -0.82%)* | 0.0047 | -0.99% (-1.15% - -0.83%)* | 0.5085 | -0.62% (-0.78% - -0.47%)* | 0.0117 | -0.53% (-0.64% - -0.41%)* |
| Oceania | 0.1445 | 0.14% (-0.09% - 0.37%) | 0.0027 | 0.23% (0.08% - 0.38%)* | 0.535 | 0.09% (-0.16% - 0.34%) | 0.0106 | 0.16% (-0.1% - 0.43%) | 0.0142 | 0.96% (0.83% - 1.08%)* | 0.0003 | 0.96% (0.83% - 1.09%)* | 0.1867 | -0.02% (-0.1% - 0.05%) | 0.004 | 0.02% (-0.05% - 0.1%) | 0.7343 | 0.73% (0.61% - 0.85%)* | 0.0153 | 0.75% (0.64% - 0.86%)* |
| South Asia | 0.1366 | 0.14% (-0.41% - 0.7%) | 0.0025 | 0.19% (-0.32% - 0.7%) | 0.2665 | 1.46% (1.31% - 1.61%)* | 0.0058 | 1.52% (1.38% - 1.66%)* | 0.0496 | 1.65% (1.26% - 2.04%)* | 0.0015 | 1.77% (1.34% - 2.19%)* | 0.2944 | -0.4% (-0.71% - -0.08%)* | 0.0063 | -0.37% (-0.73% - -0.02%)* | 0.1303 | -0.83% (-1.03% - -0.63%)* | 0.0029 | -0.71% (-0.91% - -0.51%)* |
| Southeast Asia | 0.2967 | 1.05% (0.93% - 1.18%)* | 0.0056 | 1.1% (0.99% - 1.21%)* | 0.5259 | 1.92% (1.83% - 2.01%)* | 0.011 | 1.98% (1.92% - 2.03%)* | 0.038 | 1.88% (1.81% - 1.94%)* | 0.0011 | 2.12% (2.02% - 2.21%)* | 0.1457 | 0.15% (-0.09% - 0.4%) | 0.0032 | 0.28% (0.04% - 0.53%)* | 1.0308 | -0.39% (-0.5% - -0.28%)* | 0.0221 | -0.22% (-0.41% - -0.04%)* |
| Southern Latin America | 0.5725 | 0.62% (0.53% - 0.71%)* | 0.0108 | 0.66% (0.58% - 0.74%)* | 0.8744 | 0.88% (0.64% - 1.13%)* | 0.0191 | 1% (0.78% - 1.23%)* | 0.0627 | -0.17% (-0.35% - 0.02%) | 0.002 | -0.16% (-0.32% - 0.01%) | 0.1599 | -2.99% (-3.32% - -2.67%)* | 0.0037 | -2.94% (-3.25% - -2.63%)* | 0.7491 | -0.72% (-0.97% - -0.46%)* | 0.017 | -0.56% (-0.82% - -0.31%)* |
| Southern Sub-Saharan Africa | 0.1035 | -0.65% (-1.15% - -0.15%)* | 0.0018 | -0.65% (-1.07% - -0.24%)* | 0.1731 | -0.35% (-0.76% - 0.07%) | 0.0032 | -0.5% (-0.88% - -0.12%)* | 0.1302 | -2.58% (-2.87% - -2.29%)* | 0.0034 | -2.4% (-2.68% - -2.11%)* | 0.0307 | -2.3% (-2.93% - -1.66%)* | 0.0006 | -2.46% (-2.93% - -1.99%)* | 0.3913 | -2.79% (-3.33% - -2.24%)* | 0.0075 | -2.82% (-3.3% - -2.35%)* |
| Tropical Latin America | 0.377 | 0.81% (0.45% - 1.18%)* | 0.007 | 0.81% (0.45% - 1.17%)* | 0.7993 | 0.56% (0.38% - 0.75%)* | 0.0172 | 0.73% (0.58% - 0.89%)* | 0.0518 | 0.72% (0.55% - 0.88%)* | 0.0017 | 0.83% (0.69% - 0.98%)* | 0.1172 | -3.32% (-3.62% - -3.01%)* | 0.0027 | -3.12% (-3.4% - -2.84%)* | 0.4148 | -1.06% (-1.2% - -0.93%)* | 0.0093 | -0.88% (-1.02% - -0.74%)* |
| Western Europe | 0.1947 | -0.17% (-0.31% - -0.04%)* | 0.003 | -0.68% (-0.8% - -0.56%)* | 0.4626 | 0.52% (0.42% - 0.61%)* | 0.0115 | 0.87% (0.8% - 0.94%)* | 0.0691 | -0.37% (-0.45% - -0.29%)* | 0.0021 | -0.32% (-0.41% - -0.23%)* | 0.0602 | -4.08% (-4.34% - -3.82%)* | 0.0012 | -4.33% (-4.59% - -4.08%)* | 0.1572 | -1.82% (-2.12% - -1.52%)* | 0.0041 | -1.37% (-1.55% - -1.19%)* |
| Western Sub-Saharan Africa | 0.1154 | -0.08% (-0.22% - 0.06%) | 0.0021 | -0.07% (-0.16% - 0.01%) | 0.1975 | 0.51% (0.4% - 0.61%)* | 0.004 | 0.55% (0.45% - 0.64%)* | 0.0533 | 1.16% (1.03% - 1.3%)* | 0.0018 | 1.21% (1.05% - 1.37%)* | 0.2051 | -0.1% (-0.2% - 0%)* | 0.0041 | -0.11% (-0.19% - -0.02%)* | 0.3385 | -0.05% (-0.14% - 0.03%) | 0.0075 | -0.1% (-0.21% - 0.01%) |
