# Supplementary material for: Global disease burden and trends of leukemia attributable to occupational risk from 1990 to 2019: An observational trend study
Source: Front Public Health. 2022 Nov 14;10:1015861. doi: 10.3389/fpubh.2022.1015861 (PMC9703980; doi:10.3389/fpubh.2022.1015861)
Supplement: Supplementary Table 2 — The trends of different types of leukemia attributable to occupational risk among SDI quintiles between 1990 and 2019. [file Table_2.docx]

**Table S 2.** The trends of different types of leukemia attributable to occupational risk among SDI quintiles between 1990 and 2019.

|  | **Acute lymphoid leukemia** | | **Acute myeloid leukemia** | | **Chronic lymphoid leukemia** | | **Chronic myeloid leukemia** | | **Other leukemia** | | **Leukemia** | |
| --- | --- | --- | --- | --- | --- | --- | --- | --- | --- | --- | --- | --- |
| **Location name** | **DALYs (EAPC)** | **Deaths (EAPC)** | **DALYs (EAPC)** | **Deaths (EAPC)** | **DALYs (EAPC)** | **Deaths (EAPC)** | **DALYs (EAPC)** | **Deaths (EAPC)** | **DALYs (EAPC)** | **Deaths (EAPC)** | **DALYs (EAPC)** | **Deaths (EAPC)** |
| Global | 0.78% (0.65% - 0.91%)* | 0.75% (0.68% - 0.82%)* | 0.87% (0.81% - 0.93%)* | 0.96% (0.91% - 1.01%)* | 0.66% (0.51% - 0.81%)* | 0.55% (0.43% - 0.68%)* | -0.74% (-0.89% - -0.6%)* | -0.86% (-1.03% - -0.69%)* | -1.51% (-1.7% - -1.32%)* | -1.33% (-1.48% - -1.19%)* | -0.38% (-0.58% - -0.18%)* | -0.3% (-0.45% - -0.14%)* |
| High SDI | -0.14% (-0.28% - 0.01%) | -0.36% (-0.49% - -0.22%)* | 0.23% (0.16% - 0.3%)* | 0.5% (0.44% - 0.56%)* | -0.4% (-0.59% - -0.22%)* | -0.54% (-0.76% - -0.31%)* | -3.27% (-3.45% - -3.09%)* | -3.46% (-3.63% - -3.3%)* | -1.56% (-1.69% - -1.42%)* | -1.3% (-1.49% - -1.11%)* | -0.71% (-0.82% - -0.61%)* | -0.6% (-0.64% - -0.55%)* |
| High-middle SDI | 0.77% (0.58% - 0.95%)* | 0.59% (0.44% - 0.75%)* | 0.62% (0.45% - 0.79%)* | 0.74% (0.61% - 0.86%)* | 0.87% (0.59% - 1.15%)* | 0.64% (0.36% - 0.93%)* | -1.94% (-2.26% - -1.61%)* | -1.97% (-2.37% - -1.58%)* | -1.69% (-2.04% - -1.34%)* | -1.52% (-1.7% - -1.33%)* | -0.55% (-0.81% - -0.28%)* | -0.51% (-0.74% - -0.28%)* |
| Middle SDI | 1.31% (1.16% - 1.47%)* | 1.31% (1.18% - 1.45%)* | 1.66% (1.6% - 1.72%)* | 1.71% (1.66% - 1.76%)* | 1.05% (0.74% - 1.36%)* | 1.16% (1% - 1.32%)* | -0.23% (-0.39% - -0.08%)* | -0.22% (-0.34% - -0.09%)* | -1.7% (-1.89% - -1.51%)* | -1.53% (-1.69% - -1.37%)* | -0.34% (-0.59% - -0.09%)* | -0.22% (-0.4% - -0.03%)* |
| Low-middle SDI | 0.88% (0.64% - 1.12%)* | 0.94% (0.72% - 1.15%)* | 1.67% (1.55% - 1.79%)* | 1.74% (1.63% - 1.85%)* | 1.55% (1.35% - 1.76%)* | 1.71% (1.49% - 1.94%)* | -0.24% (-0.52% - 0.04%) | -0.2% (-0.49% - 0.1%) | -0.93% (-1.09% - -0.76%)* | -0.75% (-0.87% - -0.62%)* | 0.13% (-0.05% - 0.31%) | 0.24% (0.09% - 0.38%)* |
| Low SDI | 0.98% (0.82% - 1.14%)* | 0.97% (0.83% - 1.11%)* | 1.48% (1.41% - 1.55%)* | 1.52% (1.45% - 1.58%)* | 1.84% (1.76% - 1.92%)* | 1.94% (1.87% - 2.02%)* | -0.59% (-0.69% - -0.49%)* | -0.55% (-0.64% - -0.45%)* | 0.04% (0% - 0.08%) | 0.05% (0.01% - 0.09%)* | 0.28% (0.2% - 0.37%)* | 0.33% (0.24% - 0.42%)* |
